# Effect of childbearing-age women’s family status on the health status of three generations: evidence from China

**DOI:** 10.3389/fpubh.2023.1244581

**Published:** 2023-09-14

**Authors:** Yijie Ding, Fanglei Zhong

**Affiliations:** School of Economics, Minzu University of China, Beijing, China

**Keywords:** childbearing-age women, women’s family status, health status, China family panel studies, generations

## Abstract

It is widely recognized that inequalities in social status cause inequalities in health. Women in a family often directly influence three generations–women themselves, their children and their parents -yet the effect of women’s family status on their own health status and that of the two generations before and after is not clear. Taking data from the China Family Panel Studies, this study used an ordered response model to investigate the effect of childbearing-age women’s family status on the health status of three generations. The results showed that increases in childbearing-age women’s family status improved the health status of the women themselves and their children. Unlike previous studies, however, we found that higher family status did not improve parents’ health status but decreased it. The mechanism analysis indicated that women’s family status influenced the health status of three generations through economic conditions, resource allocation, and child discipline. The results held after robustness testing. Our findings contribute to knowledge in related fields and provide theoretical support for policies that empower women.

## 1. Introduction

Health inequalities related to socioeconomic status have long been a concern, and since the 1930s, the health statuses of different socioeconomic groups in Western countries have shown systematic, multidimensional differences. Despite advancements in medical and health-related technologies and improvements to social security, the problem of health inequality has not been reduced but has even expanded in certain countries. Since the “reform and opening up” in 1978, China has faced a similar dilemma (1). Health shows imbalances across regions (2), and the unequal distribution of healthcare resources between urban and rural areas has led to a significant moderating effect of urban and rural households on individual health resources (3). In light of such phenomena, studies have investigated the direct effects of individual health inequalities caused by social status inequalities (4, 5). Studies on effect mechanisms have mainly been conducted from macro-level perspectives, including medical conditions, environmental quality, and social capital, and also from the cognitive and structural social capital dimensions of individuals (6). Fewer studies, however, have investigated the indirect health effects of socioeconomic inequalities.

Family status reflects an individual’s decision-making power in family-related matters, including access to healthcare resources and services. Childbearing-age women in a family are, on the one hand, highly vulnerable to inequitable treatment; on the other hand, they are very important in the family, often serving as the hub of the family’s daily life and assuming primary responsibility for the care of children and older adults. In terms of direct effects, the health status of a childbearing-age woman directly affects the health status of her fetus (7). Indirectly, it has been shown that when women are involved in household decision-making, all members are more likely to have access to formal healthcare; however, when the primary decision-maker is a man, only adult and adolescent males are likely to have access to formal healthcare (8). Thus, changes in the family and health status of childbearing-age women might affect the health of three generations, that is, enhancing women’s family status can improve their own health and that of other family members. This is in line with Sen’s (9) observation that “giving women a higher status is essential to achieving a better future for all.” Nevertheless, there is currently more research on the direct individual health effects of family status inequality and less research on the indirect effects, or even overall effects, on the family.

This study, therefore, aimed to examine whether women’s family status (high or low) affects their own health status and that of the two generations before and after them. To this end, we applied methods such as ordered logit models to data from the China Family Panel Studies (CFPS). The marginal contributions of this study are as follows. First, based on the phenomena of health inequality and gender inequality in China, we studied the causal relationship between women’s empowerment and their health status within the family. This can provide new paths for research on women’s development, empowerment, and well-being. Second, we expanded the scope of the analysis by considering the family as a whole and linking women with the upper and lower generations to study the effects of women’s family status. Third, we studied the heterogeneous effects of women’s family status on fathers and mothers based on gender differences. This adds new findings to the literature on gender differences in intergenerational support. It can also help governments consider gender differences in retirement support policies, balance gender interests in retirement protection, and promote social security.

The rest of this paper is organized as follows. Section 2 reviews the literature. Section 3 presents the data sources, variable setting, and descriptive statistics. Section 4 presents the results, and sections 5 and 6 present the heterogeneity and robustness tests, respectively. Section 7 discusses the findings and implications.

## 2. Literature review, theoretical mechanisms, and hypotheses

### 2.1. Literature review

#### 2.1.1. Methods for measuring women’s family status

Women’s family status refers to women’s ability and freedom to determine the allocation of family resources and choose their personal lifestyles. Its constituent elements include economic, political, educational, health, marital, and family aspects, making it a multidimensional concept (10). Current measurement methods include both single and multidimensional indicators.

Single indicators can be mainly divided into political and economic perspectives. Studies adopting a political perspective suggest that women leaders can establish images of female role models among the public and change parents’ expectations of their daughters and girls’ expectations for themselves. You and Yao (11) represented women’s political status by the proportion of female Party members. They concluded that a higher proportion of women joining the Party raises women’s political status. Specifically, they found that with a higher proportion of women joining the Party in a given year, there was a higher proportion of female live births and surviving children in the subsequent period, as well as improvements in women’s survival rights. Xue5 measured women’s status in terms of their wages and found that gender perceptions were more equal in areas where women received higher wages. Ding (12), meanwhile, measured women’s agricultural labor participation rate. Investigating differences in cultivation between South China and North China, that study found that such differences produced differences in women’s rates of participation in agricultural cultivation, thus causing differences in women’s status between South and North China. The comparative advantage of women in the South in production aspects such as rice seedling raising and transplanting improved their social status. Meanwhile, the crude nature of wheat cultivation in the North and the more physically demanding farming methods squeezed out women’s labor participation, thus lowering their social status.

Multidimensional indicators assess women’s household status in multiple dimensions. One example is the Bangladesh Demographic and Health Survey, which collects multidimensional information to assess women’s household status, including women’s and spouses’ income use, major household purchases, food purchases, food preparation, women’s healthcare, children’s healthcare, and visits to friends and relatives (13). In the present study, multidimensional indicators are used to measure women’s household status.

In summary, regardless of the approaches used, most studies suggest that enhancing women’s “value” helps advance women’s status and improve their living spaces.

#### 2.1.2. Effect of women’s family status on family members’ well-being

Women’s changing family status affects the investment of resources at their disposal within the family, which produces differences in family members’ well-being. Women’s household status is an important determinant of women’s dietary diversity and can even change the structure of the household’s diet (14). An increase in women’s decision-making power promotes protein intake by family members; this effect is more pronounced in farming households with higher incomes (15). The higher the mother’s socioeconomic status, the mother has more ability to intervene in the risk of diseases development in children, the lower the probability of diseases such as psoriasis in offspring (16).

The level of maternal education, which is an aspect of socioeconomic status, also affects children’s development–more so than fathers’ educational levels–in areas such as self-efficacy, social behavior, and interaction skills (17). Quisumbing et al. (18) found that an increase in women’s household status could also lead to increases in the share of household education expenditure.

Since there is a two-way flow of resources between the offspring and the father’s generation within the household (i.e., the phenomenon of intergenerational support), the distribution of power can also affect the well-being of the previous generation, which can manifest as differences in old-age support. Zheng and Di (19) found that women’s family status influenced intergenerational resource distribution within a family; the more power the wife has in the family, the less financial support the family has for the paternal parents. Tang (20) suggested that with the modernization of rural families, wives play an increasingly important role in the resources and welfare of maternal family members, supporting their daily living activities and sharing in the schooling of brothers and parental support; this differs from the phenomenon of daughters getting married and not assuming parental support and household responsibilities. Therefore, one focus of the present study was maternal intergenerational support—specifically, the effect of women’s family status on their parents’ health status.

#### 2.1.3. Factors influencing individual health

Factors that influence an individual’s health include genetics, age, education level, and lifestyle. As an especially important factor affecting health, aging is related to chronic diseases that can increase the risk of heart disease, among other diseases, and negatively affect health (21). In terms of educational attainment, Belo (22) suggested that a higher level of education can lead to better psychological adjustment and thus a higher sense of well-being, as well as better perceptions of aging changes. Miret (23) found a significant association between individual well-being and health status. Regarding lifestyle, Sokoya (24) found that acquired lifestyles–such as smoking, alcohol use, diet, and nutrition–can greatly determine a person’s health status. Having health insurance also affects individual health (25).

In summary, although there are many studies on women’s status, few studies have linked women’s family status with their own health status and that of the generations before and after them. Most studies indicate that an improved status can enhance women’s well-being. We do not know, however, how women’s improved family status affects their own health status and that of the generations before and after them. This is because there are numerous factors that influence health status, such as women’s family decision-making, which can affect well-being (26). There is a need, therefore, to investigate whether the improvement of women’s family status improves their own health status and that of the two generations before and after them in the context of China’s social realities. Therefore, the innovations of this paper are as follows. First, we investigate whether the improvement of women’s family status improves their own health status and that of the two generations before and after them in the context of China’s social realities. Second, to express women’s family status in terms of women’s decision-making power in the family, thus facilitating quantification using multidimensional indicators.

### 2.2. Theoretical mechanisms and hypotheses

**Figure 1:**
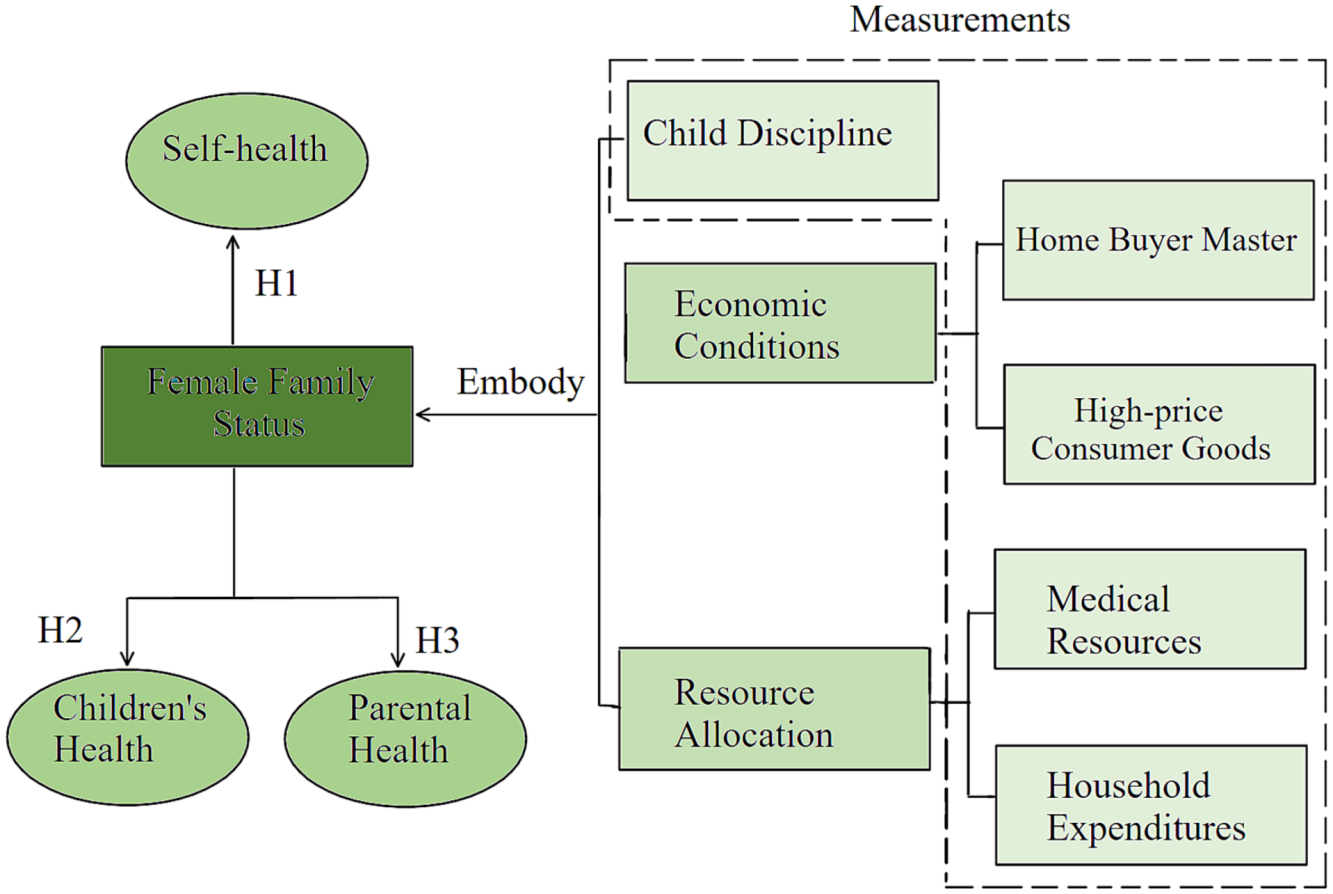
How women’s family status affects the health status of three generations.

Resource theory suggests that the relative resource holdings of each spouse in the family are key factors influencing decision-making power in the family. In the past, under more traditional gender role concepts, men held the dominant power in the family. Over time, women have become more involved in decision-making about the allocation of family resources, which could have effects on their personal health as well as that of the next two generations. The effect of childbearing-age women’s family status on the health status of individuals and different generations can be analyzed in the following ways:

1. Child discipline. Childbearing-age women with a lower family status emphasize obedience and are more likely to adopt authoritarian parenting styles, such as shouting, threatening, and physical punishment (27, 28). And when parents have positive parenting attitudes, there are usually better parental self-health ratings, parental mental health, and child health in such families. Thus, when women’s family status improved, the family functioning will also change, improving the health of both parents and children (29). Thus, when childbearing-age women’s family status improves, parent–child relationships might also change; the sense of belonging and attachment brought about by such changes could improve the coping ability and resilience of childbearing-age women in the face of difficulties (30). This in turn affects her own health status and improves her children’s psychological status, which affects their health status.
2. Economic conditions. Childbearing-age women with a high family status tend to have higher income levels and a professional status (31). They have access to better resources from society, which gives them more options for supporting their health, such as easier access to high-quality healthcare. Meanwhile, childbearing-age women with a lower family status might be financially constrained, which could negatively affect their health status. Childbearing-age women with a higher family status might also be able to provide more financial support to the next generation, which could have positive effects on their health. Financial support has critical effects on the health of children (32) and the quality of life of older adults (33). Conversely, childbearing-age women with a lower family status might not be able to provide the same level of care and support for the previous generation.
3. Resource allocation. Childbearing-age women with a higher family status usually have better financial and medical resources, which helps them provide better living environments and medical care for their family members. Childbearing-age women with a higher family status can change how they allocate family resources, such as medical resources or household goods, to themselves and their more intimate family members, such as parents and children (Figure 1).

The above discussion makes it clear that changes in women’s family status have effects on themselves and their family members. This study, therefore, modeled the effect of childbearing-age women’s family status on the health status of different family members using an ordered logit model. Three hypotheses were tested using this model. The first hypothesis (H1) concerns the effect of women’s family status on their own health status. In line with the literature, we expected to find a significant effect of women’s increased family status on their own health status. The second and third hypotheses (H2, H3) concern the effect of women’s family status on their family members’ health status, especially children and parents; likewise, we expected the former to have a significant effect on the latter. The specific hypotheses are as follows:

*H1*: Women’s family status has a significant effect on their own health status.

*H2*: Women’s family status has a significant effect on their children’s health status.

*H3*: Women’s family status has a significant effect on their parents’ health status.

## 3. Materials and methods

### 3.1. Data sources

Our data came from the CFPS, a nationwide, large-scale, multidisciplinary social tracking survey designed to reflect social, economic, demographic, educational, and health changes in China. It tracks and collects data at the individual, household, and community levels, focusing on the economic and noneconomic well-being of Chinese residents, as well as economic activity, educational attainment, family dynamics, demographic migration, and health. The CFPS covers 25 Chinese provinces, municipalities, and autonomous regions and includes all household members in the sample. Although the CFPS is a tracking survey, only the 2014 survey collected information on respondents’ intrahousehold decision-making power and related data. Therefore, we mainly used CFPS 2014 data to study the effect of childbearing-age women’s family status on the health status of three generations. Childbearing-age women are women who have reached sexual maturity but have not yet entered menopause, generally between the ages of 15 and 49. Article 6 of China’s Marriage Law states, “The age of marriage shall not be earlier than 20 years for women.” Thus, the majority of married childbearing-age women in China should be aged between 20 and 49. Therefore, women whose spouses were not deceased between the ages of 20 and 49 were selected as the sample and matched with their parents and children. After eliminating samples with serious missing data for the main variables, a final sample of 24,596 valid samples was obtained.

### 3.2. Variable settings

#### 3.2.1. Core explanatory variables: measurement of women’s household status

There are many different ways to measure women’s family status. Referring to Li and Feng (26), we measured women’s status in the family using five items mentioned above, namely, Child discipline, Home Buyer Master, High-price Consumer Goods, Medical Resources, Household Expenditures. This was done by assigning a value of 1 if the matter was “decided by the wife” and 0 if it was “decided by the husband” or “jointly negotiated by the couple.” Since it has been found that most so-called joint consultations are dominated by the husband, the five 0–1 variables were constructed, and their scores were summed to construct a composite variable reflecting women’s family status. The larger the value, the higher the status of women in the family.

#### 3.2.2. Explanatory variable: health status measurement

In the CFPS, respondents’ health status was observed by the interviewer without asking questions; the interviewer chose a value ranging from 1 to 7. The higher the value, the healthier the interviewer considered the respondent to be.

Since some of the children in the interviewed households were young and not yet able to answer the questionnaire independently, parents were invited to answer the questionnaire on their behalf. This included information about the children’s health status, which was used to obtain the health status of the children of childbearing-age women. The parent chose from values ranging from 1 to 7, with higher values indicating that the parent considered the child to be healthier. Since childbearing-age women might have more than one child, the health status scores of all children were averaged.

#### 3.2.3. Control variables

Based on the literature, we controlled for other individual characteristic variables that might affect health status, including individual’s age, education level, total personal income, household type, having health insurance, and number of children. We further included province dummy variables to control for the effects of geographical differences. The specific assignment methods were as follows: the type of household registration was 1 for nonagricultural households and 0 for agricultural households. Education level was assigned as 1–8 in descending order, with the highest level being a PhD and the lowest being illiterate/semiliterate. Having medical insurance was assigned as 1, and the absence of insurance was assigned as 0.

### 3.3. Descriptive statistics

Table 1 shows the descriptive statistics for the main variables. As we can see in the table, the mean value of Health (i.e., health status) was 5.752, and the median value was 6. This indicates that the women in the sample had a relatively good health status overall. The mean value of Decision was 1.383, and the median value was 0. This indicates that the status of women in the sample was not so good in the family, and only a small number of women had the “right to speak” in the family and the ability to participate in family decision-making. This is consistent with the reality of Chinese families, in which the husband is responsible for most family matters, and the wife assumes a supporting role.

**Table 1:** Descriptive statistics of the main variables.

Variable	*N*	Mean	Std.Dev.	Min	P50	Max
Health	24,596	5.7519	1.0483	1	6	7
Health_child	16,767	5.8194	0.9732	1	6	7
Health_mother	1,475	5.1784	1.3116	1	5	7
Health_father	1,540	5.3691	1.1647	1	5	7
Decision	24,596	1.3827	1.8784	0	0	5
Age	24,596	37.2935	7.5710	20	38	49
Age_m	1,475	56.1072	7.0897	40	56	81
Age_f	1,540	58.3476	7.6275	43	58	86
Education	24,596	2.5425	1.2512	1	3	8
Edu_m	1,475	1.9634	1.0667	1	2	6
Edu_f	1,540	2.4262	1.1495	1	2	7
Hukou	24,596	0.2154	0.4111	0	0	1
Hukou_m	1,474	0.3263	0.4690	0	0	1
Hukou_f	1,540	0.3236	0.4680	0	0	1
Childnum	24,596	1.7661	0.8199	0	2	7
Insurance	24,596	0.9175	0.2751	0	1	1
Insurance_m	1,474	0.9464	0.2253	0	1	1
Insurance_f	1,539	0.9565	0.2041	0	1	1
Income	24,596	6856.3560	14549.6100	0	0	270,000
Income_m	1,474	2347.8365	6261.6480	0	0	50,000
Income_f	1,528	8474.1571	14135.7200	0	990	96,000
Ln_Income	24,596	3.0216	4.5050	0	0	12.5062
mLn_Income	1,474	3.1988	3.7988	0	0	10.8198
fLn_Income	1,539	5.1833	4.3291	0	6.8024	11.4721

### 3.4. Ologit model

The purpose of this study was to investigate the effect of childbearing-age women’s family status on their health status after excluding other socioeconomic and demographic characteristics. Thus, this study used women’s health status with their upper and lower generations as the explanatory variables and their family status as the core explanatory variable. We established the following econometric model to identify the parameters to be estimated:


(1) $$\mathrm{Health}=\beta_{0}+\beta_{1}\times X+\beta_{2}\times \mathrm{Decision}+\mu_{i}\text{.}$$

Here, the explanatory variable of the model is Health, which represents the individual’s health status. The core explanatory variable is Decision, which represents women’s decision-making power in family matters. The control variable X includes individual’s age, education level, total personal income, household type, whether he/she has health insurance, happiness, and number of children. $\mu_{i}$ is a random error term. Given the ordered characteristics of health status, we used an ordered response model to estimate the model parameters. However, the estimated coefficients in a nonlinear model are not the marginal effects of the parameters; thus, the marginal effects of each parameter need to be calculated to better measure their effects on the explanatory variables. The large span of values for the explanatory variable Health (1–7) would make the results for marginal effects calculated based on the ordered response model more complicated, with each coefficient corresponding to a marginal effect of seven points. To facilitate the regressions, the marginal effects at Health = 6 and 7 were reported as “healthy” and “very healthy,” respectively, according to the seven-point Likert scale.

### 3.5. VIF-based multicollinearity test

When using regression, if serious multicollinearity exists in the variables, it will make it difficult to calculate the model parameter estimates, and the variance of the regression coefficients will increase with increased covariance among the variables. This will lead to the regression equation showing a significant condition. In some cases, however, the regression coefficients of independent variables that are highly correlated with the dependent variable will be eliminated when doing the significance test, and the deletion of important variables will seriously affect the accuracy of the model. Therefore, to obtain accurate regression equation coefficients and significance tests, we needed to test for and remove multicollinearity between variables. The variance inflation factor (VIF) is a measure of the severity of complex (multiple) covariance in a multiple linear regression model. It represents the ratio of the variance of the estimated regression coefficients compared to the variance when no linear correlation is assumed between the independent variables. When VIF < 10, it can be considered that there is no multicollinearity. Based on Tables 2–4, we can see that the model did not have multicollinearity problems.

**Table 2:** Multiple covariance tests for women’s status and their own health.

**Variable**	**VIF**	**1/VIF**
Decision	1.1280	0.8866
Age	1.2994	0.7696
Education	1.7607	0.5680
Hukou	1.5511	0.6447
Childnum	1.4471	0.6910
Insurance	1.0544	0.9484
Ln_income	1.1854	0.8436

**Table 3:** Multiple covariance tests for women’s status and their children’s health.

Variable	VIF	1/VIF
Decision	1.1497	0.8698
Age	1.3782	0.7256
Education	1.7292	0.5783
Hukou	1.4928	0.6699
Insurance	1.0695	0.9350
Childnum	1.5021	0.6657
Ln_income	1.1895	0.8407

**Table 4:** Multiple covariance tests for women’s status and parental health.

Variable	VIF	1/VIF
Decision	1.4141	0.7071
Health	1.1997	0.8335
Age	4.5910	0.2178
Education	2.0470	0.4885
Hukou	4.3992	0.2273
Insurance	1.1583	0.8633
Ln_income	1.3139	0.7611
Age_m	8.6415	0.1157
Edu_m	1.7794	0.5620
Hukou_m	4.2830	0.2335
Insurance_m	1.3512	0.7401
mLn_income	1.8734	0.5338
Age_f	7.0496	0.1419
Edu_f	1.4286	0.6999
hukou_f	3.7018	0.2701
Insurance_f	1.2756	0.7840
fLn_income	1.4040	0.7123

## 4. Results

### 4.1. Effect of women’s family status on their own health status

We first drew on Ferrer-i-Carbonell (34), who treated health status as a continuous variable using OLS as the baseline regression. In the OLS regression results, the estimated coefficients are the marginal effects of the corresponding parameters on the explanatory variables. Thus, a positive coefficient for Decision would indicate that family decision-making power improved women’s health status. Table 5 shows the OLS and ordered logit regression results, including the estimated coefficients of each parameter and the marginal effects corresponding to Health = 6, 7. We can see that the estimated coefficients and marginal effects of the core explanatory variable Decision were positive, and both passed the significance test at the 1% level after controlling for other variables that affect individual-level health status. In terms of marginal effects, each unit increase in Decision increases the probability of a woman’s health status being “healthy” by 0.0015% and “very healthy” by 0.0082% when other variables are held constant. This shows that there was a significant positive effect of family decision-making power on women’s health status, that is, the higher the family decision-making power of women, or the higher the family status, the better the health status of women.

**Table 5:** Effect of women’s family status on their health status.

Variable	OLS	Coefficient Estimates	Margins	
		Ologit	Health = 6	Health = 7
Decision	0.0262***	0.0445***	0.0015***	0.0082***
	(0.0036)	(0.0066)	(0.0002)	(0.0012)
Age	0.0018*	0.0043**	0.0010**	0.0010**
	(0.0010)	(0.0018)	(0.0001)	(0.0003)
Education	0.0418***	0.0800***	0.0028***	0.0146***
	(0.0070)	(0.0127)	(0.0005)	(0.0024)
Hukou	0.0322*	0.0344	0.0011	0.0063
	(0.0193)	(0.0362)	(0.0013)	(0.0067)
Childnum	−0.0864***	−0.1520***	−0.0053***	−0.0279***
	(0.0096)	(0.0168)	(0.0006)	(0.0030)
Insurance	0.1560***	0.2770***	0.0095***	0.0507***
	(0.0243)	(0.0439)	(0.0016)	(0.0085)
Ln_income	−0.0027*	−0.0040	−0.0001	−0.0007
	(0.0016)	(0.0029)	(0.0001)	(0.0005)
Provincial fixed effects	YES	YES	YES	YES
Adjusted *r*^2^/*r*^2^	0.0699	0.0296	0.0296	0.0296
Observations	24,596	24,596	24,596	24,596

(*) are used to indicate significant differences between levels. In general, the higher the number of asterisks, the higher the significance of the difference is indicated. (*) indicates a value of p less than 0.05, (**) indicate a value of p less than 0.01, and (***) indicate a value of p less than 0.001.

Childbearing-age women with a high family status usually have a higher level of education. They can acquire health-related knowledge directly through education and develop self-awareness of health, which in turn lead to various health-promoting behaviors (35). Second, individuals with higher education levels can expand their social networks through participation in cultural activities, which can expand the channels for acquiring health knowledge (36). Therefore, childbearing-age women with a higher family status will acquire health knowledge efficiently owing to their higher education level, and they will tend to pay more attention to their health and health behaviors such as diet and exercise. Meanwhile, childbearing-age women with a lower family status might acquire health knowledge less efficiently owing to their lower education level, which might lead to a lower health status.

### 4.2. Effect of women’s family status on their children’s health status

We investigated the relationship between women’s family status and their children’s health status in a similar manner. Children’s health status was averaged across childbearing-age women because they had different numbers of children. To facilitate the subsequent analysis of marginal effects while rounding off the health status of children, we named the indicator Health_childr.

Table 6 shows the OLS and Ologit regression results for women’s family status and their children’s health status, including the estimated coefficients of each parameter and the marginal effects corresponding to Health = 6, 7. The estimated coefficients and marginal effects of the core explanatory variable Decision were positive, and both passed the significance test at the 1% level after controlling for other variables that affect individual-level health status. In terms of marginal effects, each unit increase in Decision increased the probability of a female child’s health status being “healthy” by about 0.0003% and “very healthy” by about 0.0042%. Thus, there was a significant positive effect of women’s family decision-making power on the health status of children within the family. In other words, the higher the decision-making power of women, or the higher the family status, the better the health status of children in the family. There could be several reasons for this.

**Table 6:** Effect of women’s family status on their children’s health status.

Variable	OLS	Coefficient Estimates	Margins	
		Ologit	Health = 6	Health = 7
Decision	0.0152***	0.0223**	0.0003**	0.0042**
	(0.0043)	(0.0087)	(0.0001)	(0.0017)
Age	−0.0080***	−0.0129***	−0.0002***	−0.0025***
	(0.0013)	(0.0026)	(0.0001)	(0.0005)
Education	0.0556***	0.1170***	0.0015***	0.0223***
	(0.0078)	(0.0156)	(0.0003)	(0.0030)
Hukou	0.0402***	0.0807***	0.0010***	0.0154***
	(0.0111)	(0.0224)	(0.0003)	(0.0043)
Childnum	0.0948***	0.1320**	0.0017**	0.0251**
	(0.0267)	(0.0525)	(0.0007)	(0.0100)
Insurance	−0.0646***	−0.1510***	−0.0020***	−0.0288***
	(0.0112)	(0.0213)	(0.0004)	(0.0041)
Ln_income	−0.0046***	−0.0064*	−0.0001*	−0.0012*
	(0.0018)	(0.0035)	(0.0001)	(0.0007)
Provincial fixed effects	YES	YES	YES	YES
Adjusted *r*^2^/*r*^2^	0.0866	0.0377	0.0377	0.0377
Observations	16,767	16,767	16,767	16,767

(*) are used to indicate significant differences between levels. In general, the higher the number of asterisks, the higher the significance of the difference is indicated. (*) indicates a value of p less than 0.05, (**) indicate a value of p less than 0.01, and (***) indicate a value of p less than 0.001.

First, in terms of resource allocation within the family, some studies have shown that women are more concerned about the health of their children. For example, some physiological factors lead to women being more likely to be involved in caring for the health of their children. Breastfeeding, for example, might motivate mothers to pay more attention to their own and their children’s diet and nutrition. In addition, women are mostly involved in family care and child-rearing, giving them more direct knowledge of and responsibility for the development and health of their children. Therefore, when a woman’s family status is higher, she will likely allocate more family resources to her child to support his/her development; thus, the child’s health will be better.

Second, with regard to pregnancy, the health status of childbearing-age women can seriously affect the health status of the fetus. Thus, childbearing-age women need to pay more attention to their nutrition, exercise, and health management during pregnancy. Some studies have shown, for example, that abnormal thyroid function in pregnant women can lead to mental impairment and slow growth of the fetus (7). Women of reproductive age with a high family status are more likely to receive good maternal healthcare, thus helping to ensure healthy fetal growth. Women with a low family status might not receive the same maternal healthcare, which might affects fetal health.

In terms of parenting knowledge and behavior, childbearing-age women with a high family status usually have more parenting knowledge and resources and are able to provide better nutrition, education, and care for their children. Women with a lower family status, meanwhile, might not be able to provide the same for their children because they lack the relevant knowledge and resources.

Finally, in terms of family environment, childbearing-age women with a high family status tend to be able to create a more harmonious and safe family environment for their children, which contributes to their healthy development. Women with a lower family status, meanwhile, might not be able to provide the same family environment.

### 4.3. Effect of women’s family status on their parents’ health status

Table 7 shows the OLS and Ologit regression results for women’s family status and their parents’ health status, including the estimated coefficients of each parameter and the marginal effects corresponding to Health = 6, 7. The estimated coefficients and marginal effects of the core explanatory variable Decision were negative, and both passed the significance test at the 1% level after controlling for other variables that affect individual-level health status. In terms of marginal effects, each unit increase in Decision reduced the probability of a female parent’s health status being “healthy” by about 0.0038% and “very healthy” by about 0.0138%. The current model of older adults care in China is mainly family care, and such care is mainly provided by female members of the family, especially daughters and daughters-in-law. In the past, however, under the dual influence of patriarchy and the “superiority first” principle of family resource allocation, it was nearly impossible for women to allocate resources to their own parents. With modernization, however, women’s economic independence has increased, and the patriarchal culture has weakened; the phenomenon of daughters’ retirement has gradually emerged, and women can now allocate more family resources to their own parents (37). Yet, this increase in resource allocation does not necessarily have a positive effect on parental health, and thus, an increase in women’s family status does not necessarily have a positive effect on parental health. There are several possible reasons for this.

**Table 7:** Effect of women’s family status on their parents’ health status.

Variable	OLS	Coefficient Estimates	Margins	
		Ologit	Health_p = 6	Health_p = 7
Decision	−0.0664***	−0.1700***	−0.0038***	−0.0138***
	(0.0167)	(0.0468)	(0.0011)	(0.0039)
Health	0.6605***	1.8465***	0.0410***	0.1500***
	(0.0243)	(0.0864)	(0.0038)	(0.0080)
Age	0.0201***	0.0330*	0.0007*	0.0027*
	(0.0067)	(0.0173)	(0.0004)	(0.0014)
Education	0.1667***	0.3945***	0.0088***	0.0321***
	(0.0205)	(0.0567)	(0.0017)	(0.0044)
Hukou	−0.1058	−0.0972	−0.0022	−0.0079
	(0.0754)	(0.2275)	(0.0051)	(0.0185)
Insurance	−0.1639**	−0.4663***	−0.0103***	−0.0379***
	(0.0662)	(0.1700)	(0.0039)	(0.0137)
Ln_income	−0.0085**	−0.0251**	−0.0006**	−0.0020**
	(0.0041)	(0.0109)	(0.0003)	(0.0009)
Age_m	−0.0041	−0.0462*	−0.0010*	−0.0038*
	(0.0110)	(0.0241)	(0.0005)	(0.0020)
Edu_m	0.0602***	0.1435***	0.0032**	0.0117***
	(0.0205)	(0.0533)	(0.0013)	(0.0043)
Hukou_m	−0.2526***	−0.8530***	−0.0189***	−0.0694***
	(0.0769)	(0.2320)	(0.0054)	(0.0191)
Insurance_m	0.3526***	1.2490***	0.0277***	0.1020***
	(0.1163)	(0.3282)	(0.0082)	(0.0255)
mLn_income	0.0098	0.0197	0.0004	0.0016
	(0.0060)	(0.0150)	(0.0003)	(0.0012)
Age_f	−0.0194*	−0.0033	−0.0001	−0.0003
	(0.0099)	(0.0209)	(0.0005)	(0.0017)
Edu_f	−0.0101	−0.0310	−0.0007	−0.0025
	(0.0209)	(0.0545)	(0.0012)	(0.0044)
Hukou_f	0.1605**	0.5882***	0.0131***	0.0479***
	(0.0681)	(0.2000)	(0.0047)	(0.0162)
Insurance_f	0.0364	0.3306	0.0073	0.0269
	(0.1090)	(0.2638)	(0.0058)	(0.0216)
fLn_income	0.0172***	0.0506***	0.0011***	0.0041***
	(0.0045)	(0.0127)	(0.0003)	(0.0010)
Provincial fixed effects	YES	YES	YES	YES
Adjusted *r*^2^/*r*^2^	0.5760	0.2214	0.2214	0.2214
Observations	1,474	1,474	1,474	1,474

(*) are used to indicate significant differences between levels. In general, the higher the number of asterisks, the higher the significance of the difference is indicated. (*) indicates a value of p less than 0.05, (**) indicate a value of p less than 0.01, and (***) indicate a value of p less than 0.001.

First, Song (38) found that increases in financial and health care support by children reduce the health status of the older adults to a certain extent. That’s because the older adults might also experience psychological burdens as a result of help from their children, which might lead to poor health status. Some studies have shown that aid received from children increase depression among parents, and the amount of aid exchanged is positively related to depression, which are interpreted in terms of individuals’ perception of a loss of independence in old age (39). Intrahousehold decision-making power affects the level of financial support and care given to the older adults. Therefore, women with a higher family status are likely to reduce the health status of the older adults because they can give them more financial support and care.

Second, Wu (40) found that caring for grandchildren could have positive effects on the physical and mental health of middle-aged and older adults in both urban and rural areas, especially middle-aged women and rural middle-aged people, mainly through increased social interaction, exercise, and net transfers from children. Since family decision-making power includes disciplining children, women with higher family decision-making power might increase the amount of time they spend personally caring for their children, thus decreasing the amount of time their parents spend caring for them and thus lowering their parents’ health status.

Confucian filial piety is an important aspect of traditional Chinese culture. Filial piety concerns serving and supporting one’s parents. This has become the ideological basis for family aging in China (41). Thus, the current model of aging in China is mainly family aging, while care for the older adults in daily life is mainly undertaken by female family members, especially daughters and daughters-in-law (42). As noted earlier, with modernization, the patriarchal culture has weakened, and the phenomenon of daughters’ old-age care has gradually emerged. With the growing problem of population aging in China, coupled with the one-child policy of previous years, many women face the dual responsibility of potentially caring for their parents and in-laws in the future. This means that the burden of care for adult women will become increasingly heavy. Thus, although women’s family status has improved, they are unable to break away from their traditional responsibility for supporting the older adults, but they must also participate in family decision-making and other matters that women did not traditionally participate in. Although women’s improved family status helps them support their parents financially, they might neglect their parents’ health owing to psychological burden and other factors.

The relative power of the couple within the family is also a key factor affecting the health status of women and their related generations, suggesting that status within the family also determines the ability to access resources. The family is the starting point for access to resources. In the current environment that emphasizes women’s rights, women’s status needs to be improved in terms of not only the macro level of social development and progress (e.g., equal pay for equal work) but also the micro level of the family. In addition, the effects of most control variables in our model on individual health status were consistent with those reported in existing studies, thus ensuring the reasonableness of the selection of control variables.

### 4.4. Heterogeneity test

Owing to differences in social attitudes and cultural practices, older men and older women respond differently to intergenerational support for their children. Wang (43), for example, found that fathers significantly increased their risk of death by not living with their children, but mothers significantly lowered their risk of death based on the perspective of intergenerational cohabitation. Thus, there might be gender differences in the effect of women’s family status changes on parental health status. To further investigate variability in the effect of women’s family status on female parents, we grouped the samples separately according to parental gender. We then used an Ologit model to test the effect of women’s family decision-making power on their well-being.

As shown in Tables 8 9, women’s family status had a significant negative effect on the health status of both fathers and mothers. The estimated coefficients of each parameter and the marginal effects corresponding to Health = 6, 7 showed that the negative effect was higher for mothers than for fathers. As mentioned earlier, women might change their financial support for their parents and the time they spend caring for their grandchildren because of changes in family status. Thus, as women’s family status improves, both parents receive more financial support, but mothers might experience a greater psychological burden than fathers, resulting in a more pronounced decline in their health status. Mothers might also be more sensitive to decreases in time spent caring for their grandchildren.

**Table 8:** Effect of women’s family status on their mothers’ health status.

Variable	OLS	Coefficient Estimates	Margins	
		Ologit	Health_m = 6	Health_m = 7
Decision	−0.0783***	−0.1785***	−0.0086***	−0.0191***
	(0.0191)	(0.0422)	(0.0021)	(0.0045)
Health	0.6985***	1.5086***	0.0723***	0.1610***
	(0.0308)	(0.0809)	(0.0045)	(0.0089)
Age	0.0241***	0.0444**	0.0021**	0.0048**
	(0.0090)	(0.0191)	(0.0009)	(0.0020)
Education	0.2135***	0.3537***	0.0170***	0.0379***
	(0.0293)	(0.0625)	(0.0033)	(0.0064)
Hukou	0.0365	0.1976	0.0095	0.0211
	(0.0923)	(0.1992)	(0.0095)	(0.0214)
Insurance	−0.1763**	−0.3229*	−0.0155*	−0.0345*
	(0.0794)	(0.1792)	(0.0086)	(0.0190)
Ln_income	0.0007	0.0055	0.0003	0.0006
	(0.0056)	(0.0122)	(0.0006)	(0.0013)
Age_m	−0.0197**	−0.0321*	−0.0015*	−0.0034**
	(0.0079)	(0.0164)	(0.0008)	(0.0017)
Edu_m	0.0654**	0.1390**	0.0067**	0.0149**
	(0.0270)	(0.0564)	(0.0028)	(0.0059)
Hukou_m	−0.2267**	−0.5668***	−0.0272***	−0.0606***
	(0.0942)	(0.2044)	(0.0099)	(0.0220)
Insurance_m	0.3878***	1.0313***	0.0494***	0.1100***
	(0.1500)	(0.3313)	(0.0166)	(0.0344)
Lnincome_m	0.0028	0.0005	0.0001	0.0001
	(0.0076)	(0.0152)	(0.0007)	(0.0016)
Provincial fixed effects	YES	YES	YES	YES
Adjusted *r*^2^/*r*^2^	0.4778	0.2115	0.2115	0.2115
Observations	1,474	1,474	1,474	1,474

(*) are used to indicate significant differences between levels. In general, the higher the number of asterisks, the higher the significance of the difference is indicated. (*) indicates a value of p less than 0.05, (**) indicate a value of p less than 0.01, and (***) indicate a value of p less than 0.001.

**Table 9:** Effect of women’s family status on their fathers’ health status.

Variable	OLS	Coefficient Estimates	Margins	
		Ologit	Health_f = 6	Health_f = 7
Decision	−0.0505***	−0.1060**	−0.0041**	−0.0111**
	(0.0180)	(0.0474)	(0.0018)	(0.0050)
Health	0.6361***	1.6357***	0.0633***	0.1708***
	(0.0286)	(0.0855)	(0.0042)	(0.0075)
Age	0.0134	0.0180	0.0007	0.0019
	(0.0086)	(0.0159)	(0.0006)	(0.0017)
Education	0.1211***	0.2928***	0.0113***	0.0306***
	(0.0209)	(0.0501)	(0.0022)	(0.0051)
Hukou	−0.0790	0.0003	0.0001	0.0001
	(0.0830)	(0.2126)	(0.0082)	(0.0222)
Insurance	−0.1672**	−0.4814***	−0.0186***	−0.0503***
	(0.0669)	(0.1720)	(0.0068)	(0.0176)
Ln_income	−0.0119**	−0.0280**	−0.0011**	−0.0029**
	(0.0048)	(0.0112)	(0.0004)	(0.0012)
Age_f	−0.0198***	−0.0331***	−0.0013***	−0.0035***
	(0.0066)	(0.0120)	(0.0005)	(0.0013)
Edu_f	0.0298	0.0145	0.0006	0.0015
	(0.0208)	(0.0461)	(0.0018)	(0.0048)
Hukou_f	−0.1048	−0.4024**	−0.0156**	−0.0420**
	(0.0837)	(0.1997)	(0.0079)	(0.0208)
Insurance_f	0.1172	0.3686	0.0143	0.0385
	(0.1243)	(0.2870)	(0.0111)	(0.0299)
fLn_income	0.0253***	0.0622***	0.0024***	0.0065***
	(0.0055)	(0.0133)	(0.0005)	(0.0014)
Provincial fixed effects	YES	YES	YES	YES
Adjusted *r*^2^/*r*^2^	0.4797	0.2446	0.2446	0.2446
Observations	1,539	1,539	1,539	1,539

(*) are used to indicate significant differences between levels. In general, the higher the number of asterisks, the higher the significance of the difference is indicated. (*) indicates a value of p less than 0.05, (**) indicate a value of p less than 0.01, and (***) indicate a value of p less than 0.001.

### 4.5. Robustness tests: replace explanatory variable

In addition to the indicators used above, we also used female household financial power as a proxy indicator of women’s household status. The question “Who is the household financial decision-maker?” was evaluated. Since household financial management is only a part of household decision-making, this question cannot accurately and comprehensively measure women’s household status but may only reflect it to a certain extent. The regression results showed that the effect of women’s control over family finances on women’s health status and the health status of their next generation was consistent with the previous results, and all were significant at the 1% level (Tables 10–12).

**Table 10:** Robustness tests of the effect of women’s family status on their health.

Variable	OLS	Coefficient Estimates	Margins	
		Ologit	Health = 6	Health = 7
Finance	0.1169***	0.1835***	0.0063***	0.0336***
	(0.0137)	(0.0252)	(0.0009)	(0.0046)
Control variables	YES	YES	YES	YES
Provincial fixed effects	YES	YES	YES	YES
Adjusted *r*^2^/*r*^2^	0.0706	0.0297	0.0297	0.0297
Observations	24,596	24,596	24,584	24,584

(*) are used to indicate significant differences between levels. In general, the higher the number of asterisks, the higher the significance of the difference is indicated. (*) indicates a value of p less than 0.05, (**) indicate a value of p less than 0.01, and (***) indicate a value of p less than 0.001.

**Table 11:** Robustness tests of the effect of women’s family status on their children.

Variable	OLS	Coefficient Estimates	Margins	
		Ologit	Health_childr = 6	Health_childr = 7
Finance	0.0555***	0.0762**	0.0010**	0.0145**
	(0.0161)	(0.0323)	(0.0005)	(0.0062)
Control variables	YES	YES	YES	YES
Provincial fixed effects	YES	YES	YES	YES
Adjusted *r*^2^/*r*^2^	0.0865	0.0376	0.0376	0.0376
Observations	16,767	16,767	16,767	16,767

(*) are used to indicate significant differences between levels. In general, the higher the number of asterisks, the higher the significance of the difference is indicated. (*) indicates a value of p less than 0.05, (**) indicate a value of p less than 0.01, and (***) indicate a value of p less than 0.001.

**Table 12:** Robustness tests of the effect of women’s family status on their parents.

Variable	OLS	Coefficient estimates	Margins	
		Ologit	Health_p = 6	Health_p = 7
Finance	−0.3174***	−0.7286***	−0.0164***	−0.0591***
	(0.0695)	(0.1687)	(0.0040)	(0.0140)
Control variables	YES	YES	YES	YES
Provincial fixed effects	YES	YES	YES	YES
Adjusted *r*^2^/*r*^2^	0.5779	0.2224	0.2224	0.2224
Observations	1,474	1,474	1,474	1,474

(*) are used to indicate significant differences between levels. In general, the higher the number of asterisks, the higher the significance of the difference is indicated. (*) indicates a value of p less than 0.05, (**) indicate a value of p less than 0.01, and (***) indicate a value of p less than 0.001.

## 5. Conclusion and discussion

Gender inequality still widely exists in developing regions such as South Asia, the Middle East, and North Africa. In these countries, women generally have less access to resources than men. In China, since the reform and opening up, women have been further emancipated, and their rights to survive have been continuously improved. Women’s good health reflects greater equality in gender relations. Moreover, with socioeconomic development, women’s human capital is playing an increasingly important role, and women’s health also reflects good or bad human capital. Improving women’s family status and health is beneficial for the overall development of the country. Our results showed that the improvement of women’s family status had a significant positive effect on their health status. This can provide new paths for research on women’s development, empowerment, and well-being.

We also found that the higher the family status of women, the better their children’s health status. This could be because women’s improved health status leads to better nutritional intake and medical resources during pregnancy, which in turn affects fetal health. It might also be because women improve their children’s health by changing the way they allocate family resources and using health knowledge. Our results suggest that improving women’s living environment is beneficial not only for the health of the present generation but also for that of the next generation. This leads to new paths for enhancing children’s well-being.

We found a significant negative effect of women’s improved family status on their parents’ health status. This negative effect was greater for mothers than fathers and might include psychological burden, time spent caring for grandchildren, and so on. This adds new findings to the literature on gender differences in intergenerational support. Moreover, as a result of population aging and the one-child policy of the past, many women will face the possibility of caring for both their parents and in-laws in the future, which means the care burden of adult women will become increasingly heavy. Therefore, studying the influence of female family members on the health status of the older generation can also provide suggestions for how to reduce the burden of family aging and improve the well-being of the older adults.

Some studies have shown that parental health is positively related to co-residence and the socioeconomic status of children in Korea. In Europe and the United States, however, the situation is just the opposite (44). Therefore, the influence of women’s family status on the health status of their parents needs to be further analyzed in the light of pension policies and social culture in different countries and regions. Although this study found that the promotion of women’s family status is an important pathway to enhance the well-being of family members, especially women themselves, women’s children and women’s parents, many aspects still need to be improved. First, the sample of this study was selected only from the Chinese region, which has some geographical limitations. Second, there are control variables that may have been missed from this paper. Finally, although this paper confirms that women’s family status affects the health of women’s fathers and mothers to different degrees, the mechanisms responsible for the differences need to be explored in greater depth.

## 6. Policy recommendations

In summary, women’s family status has a significant effect on their own health status and that of their next generation, mainly through social and family support, health knowledge, medical resources, and economic conditions. Regarding health improvement, improving childbearing-age women’s family status can improve their own health status and that of the next generation. Therefore, first, policies should be introduced to help improve childbearing-age women’s status in the family and enable them to play a greater role in family decision-making by promoting gender equality, raising their education level, and providing employment opportunities. Second, we should encourage families, communities, and government departments to establish mechanisms to enhance social support and provide diverse support and assistance to childbearing-age women to reduce the pressures they face. Third, we should popularize health knowledge and promote healthy behaviors through education and guide childbearing-age women to develop good living habits, such as a good diet and moderate exercise, so that they can pay more attention to their own health and that of their families. Fourth, we need to optimize medical resources and improve grassroot medical facilities to provide childbearing-age women with convenient, high-quality medical services and ensure that they receive timely, effective treatment when needed. Fifth, through policies such as poverty alleviation and social security, we can improve the economic status of families and create better living environments and conditions for childbearing-age women. By implementing measures to improve the family status and living conditions of childbearing-age women, their health status can be effectively improved, thus promoting the health and development of society as a whole.

With regard to older adults care, the reason women’s status decreases parents’ health is related to the traditional Chinese concept of “family old-age care.” Further, the responsibility for supporting the older adults mainly falls on women. Although women’s improved status in the family allows them to provide financial support to their parents, they might not provide spiritual comfort, causing their parents’ health to decline owing to psychological burdens and other reasons. Therefore, policies should be introduced to provide more social support for the older adults, transform family care into social care, and change social attitudes in order to free women from traditional burdens so that men and women can support the older adults together.

## Data availability statement

Publicly available datasets were analyzed in this study. This data can be found at: http://www.isss.pku.edu.cn/cfps/.

## Ethics statement

The studies involving humans were approved by Biomedical Ethics Committee, Peking University. The studies were conducted in accordance with the local legislation and institutional requirements. The participants provided their written informed consent to participate in this study.

## Author contributions

YD wrote the original draft. FZ and YD revised and reviewed the article. FZ supervised the article. All authors contributed to the article and approved the submitted version.

## Funding

This work was supported by the National Key R&D Program of China (2022YFC3800705), Youth Innovation Promotion Association of Chinese Academy of Sciences (Grant No. 2020424).

## Conflict of interest

The authors declare that the research was conducted in the absence of any commercial or financial relationships that could be construed as a potential conflict of interest.

## Publisher’s note

All claims expressed in this article are solely those of the authors and do not necessarily represent those of their affiliated organizations, or those of the publisher, the editors and the reviewers. Any product that may be evaluated in this article, or claim that may be made by its manufacturer, is not guaranteed or endorsed by the publisher.
